# A High-Throughput Neurosphere-Based Colony Formation Assay to Test Drug and Radiation Sensitivity of Different Patient-Derived Glioblastoma Lines

**DOI:** 10.3390/cells13231995

**Published:** 2024-12-03

**Authors:** Manoj Kumar, Lauren C. Nassour-Caswell, Hasan Alrefai, Joshua C. Anderson, Taylor L. Schanel, Patricia H. Hicks, Rex Cardan, Christopher D. Willey

**Affiliations:** Department of Radiation Oncology, The University of Alabama at Birmingham, Birmingham, AL 35249, USA; manokuma4575@uabmc.edu (M.K.); nassour@uab.edu (L.C.N.-C.); ha0011@uab.edu (H.A.); janders7@uab.edu (J.C.A.); tschanel@uab.edu (T.L.S.); hicksp@uab.edu (P.H.H.); rcardan@uabmc.edu (R.C.)

**Keywords:** colony formation assay, clonogenic assay, radiation sensitivity, glioblastoma, patient-derived cancer cells, brain tumor initiating cells

## Abstract

The gold standard assay for radiation response is the clonogenic assay, a normalized colony formation assay (CFA) that can capture a broad range of radiation-induced cell death mechanisms. Traditionally, this assay relies on two-dimensional (2D) cell culture conditions with colonies counted by fixing and staining protocols. While some groups have converted these to three-dimensional (3D) conditions, these models still utilize 2D-like media compositions containing serum that are incompatible with stem-like cell models such as brain tumor initiating cells (BTICs) that form self-aggregating spheroids in neural stem cell media. BTICs are the preferred patient-derived model system for studying glioblastoma (GBM) as they tend to better retain molecular and phenotypic characteristics of the original tumor tissue. As such, it is important that preclinical radiation studies should be adapted to BTIC conditions. In this study, we describe a series of experimental approaches for performing CFA experiments with BTIC cultures. Our results indicate that serum-free clonogenic assays are feasible for combination drug and radiation testing and may better facilitate translatability of preclinical findings.

## 1. Introduction

Glioblastoma (GBM) is an aggressive malignant brain tumor with limited median survival (~1.5 years) following safe surgical resection, concurrent temozolomide treatment, and radiation therapy [1,2]. While there are a small number of additional approved therapies, like lomustine, carmustine, bevacizumab, and tumor treatment fields (TTF; Optune device by Novocure) [3,4,5], outcomes remain poor [6]. Considerable effort is being devoted to identifying novel treatments for this disease, but clinical success has been difficult to achieve.

Although a large number of drugs may work well at targeting GBM cells in vitro, these drugs frequently do not work efficiently in vivo or at a clinical level. A major stumbling block for the field is related to therapeutic delivery challenges, including bioavailability, solubility and biostability for conventional drug delivery approaches. For addressing these problems, new drug delivery systems like nanoparticle-based drug delivery system or other biomaterial-based drug delivery system are being studying extensively [7].

Apart from new drug delivery approaches, scientists are also exploring new therapies like photothermal therapy [8], FLASH radiation therapy [9], and immunotherapy [10] for the treatment of different cancer models like GBM. Recently, Nozhat et al. produced Fe_3_O_4_ and SiO_2_-based nanoparticles loaded with temozolomide and finally coated with platelet membrane (Fe_3_O_4_ @SiO_2_/TMZ@PLTM) to make them biocompatible. They showed in vitro evidence for synergy between drug and photothermal effect in GBM [8]. FLASH radiation (FLASH-RT) is an innovative strategy utilizing an ultra-high dose rate of radiation (>40 Gy/s) to treat various cancer types in order to minimize normal tissue toxicity compared to the conventional low dose rate of radiation (CONV-RT) [9]. There has also been considerable interest in targeting the tumor immune microenvironment in GBM. GBM cells secrete various chemokines like TGF-β, IL10, and prostaglandin E2 (PGE-2). These secretary molecules can work as immunosuppressors [11]. Cytokines like IFN-γ, TNF-α, IL-2, and IL-12 are pro-inflammatory molecules that some have proposed as potential therapeutics in GBM [12,13,14]. While immune checkpoint blockade [15] and chimeric antigen receptors T-cell therapy [10,16] have shown some promise, these approaches have had limited clinical utility.

Since radiation treatment of GBM is a near-universally used therapy, identifying novel drug therapies to use in combination with radiation has high potential value. The clonogenic assay, a colony formation assay (CFA) normalized to sham irradiation (so-called plating efficiency), is the gold standard assay for in vitro preclinical radiation sensitivity testing. However, traditional clonogenic assays are performed in two-dimensional (2D) cell culture using fixing and staining protocols that do not easily transfer to preferred preclinical GBM models that are devoid of serum with three-dimensional (3D) spheroid cultures, commonly called “neurospheres”. Neurosphere cultures grown in defined media based on neural stem cell media formulations, better preserve tumor cell biology and phenotype, and can form in vivo tumors with relatively low cell numbers. These brain tumor initiating cell (BTIC) culture techniques are now standard approaches in GBM research but have been difficult to use in clonogenic assays.

The main CFA techniques that have been reported for BTIC/neurosphere assays include agarose-based colony assay, adherent cell colony assay, Alvetex plate-based colony assay, ClonoScreen3D assay, and extreme limiting dilution assay (ELDA) [17,18,19,20,21]. Each CFA approach has some advantages and some limitations. Historically, CFA techniques use 6- or 12-well plates or 35 mm dishes to generate colonies for adherent cells. Scaffold- and matrix-based CFA techniques are compatible with neurosphere cultures but suffer from tedious setup or costly reagents, particularly when using larger-volume culture dishes. We have developed a new spheroid/neurosphere-based high-throughput colony formation assay (HT-CFA) in 96-well plates which gives us freedom to use multiple drug doses and drug combinations in a single plate. We provide details for both live cell dye staining and a fixing and staining protocol allowing for delayed analyses. We also provide examples of specialized variations including longitudinal assessment after fractionated radiation regimens, FLASH proton irradiation, and varying spheroid size handling.

## 2. Materials and Methods

### 2.1. Materials

Serum-free media was based on neural stem cell media (PDX media) which is DMEM/F12 50/50 (Corning Ref # 16-405-CV, Manassas, VA, USA) supplemented with B27 (Gibco Ref # 17504-044, Grand Island, NY, USA), 100 Units/mL penicillin + 100 μg/mL streptomycin (Corning Ref # 30-002-CI, Manassas, VA, USA), 1 mM sodium pyruvate (Corning Ref # 25-000-CI, Manassas, VA, USA), 20 ng/mL EGF (Gibco Ref # PHG0311, Carlsbad, CA, USA), and 20 ng/mL FGF (Gibco Ref # PHG0261, Carlsbad, CA, USA). Plasticware was clear-bottom black-wall 96-well plates (Corning Ref # 3603, Kennebunk, ME, USA); Imaging was performed using Cytation 5 imager (Agilent, Santa Clara, CA, USA). Fixation was performed using 4% paraformaldehyde (Thermo Scientific Cat. No. J61899.AP, Ward Hill, MA, USA). Cellular dyes included Calcein AM (Corning Ref # 354216, Bedford, MA, USA) and wheat germ agglutinin (WGA) conjugated with Alexa Fluor 488 (ThermoFisher Ref # W11261, Eugene, OR, USA). Accutase (Corning Ref # 25-058-CI, Manassas, VA, USA) was used to dissociate cells. PDX cells were obtained from either the UAB Brain Tumor Animal Models core as previously described [22] or directly from the Mayo Clinic’s PDX Repository under a Material Transfer Agreement. Some of the PDX cells were infected with mCherry or NLS mCherry lentivirus for stable fluorochrome expression [23]. While animal studies are not shown in this manuscript, the maintenance of the PDX lines was performed in mice. The UAB IACUC approved this project (Approval Animal Project Number: IACUC 21435; Renewal Approved 11 July 2023).

### 2.2. Lentiviral Particle Production and Stable Fluorochrome Expressing PDX Line Preparation

Lentiviral particles were produced using cancer stem cell 293T cells (CSC293Ts) [23] by transfecting pMD2.G (Addgene, Plasmid #12259, Watertown, MA, USA), psPAX2 (Addgene, Plasmid #12260, Watertown, MA, USA), and NLS-mCherry vector(vectorbuilder) or whole cell mCherry (Addgene plasmid # 36084, Watertown, MA, USA) at a ratio of 1:3:4. In brief, we transfected CSC293Ts in a 60 mm culture dish (70% confluency) using lipofectamine 3000 reagent (Invitrogen, Cat.#L3000015, Carlsbad, CA, USA) according to the manufacturer’s protocol. A Total of 5 μg DNA of pMD2.G: psPAX2: NLS-mCherry plasmid or whole-cell mCherry plasmid at a ratio of 1:3:4 was used for transfection. After 16 h of transfection, the media was replaced with fresh PDX media. Viral particle media was collected after 24 h and 48 h of media replacement and stored as 1 mL aliquots t −80 °C. To create stable mCherry or NLS mcherry PDX lines, 1 mL of desired viral particle-containing media was added to 24 h grown PDX line (~100,000 cells) on a geltrex-coated plate. After 2 days of infection, PDX lines were treated with 0.5 μg/mL puromycin PDX media for 2 days then transferred into fresh PDX media. After culturing these cells in bigger volumes, positive cells were sorted using the mCherry channel on a flow cytometer.

### 2.3. Colony Formation Assay and Clonogenic Survival Assay

Neurosphere cultures in serum-free PDX media at 37 °C in a humidified 5% CO_2_ incubator were used to expand cell numbers for CFA testing. Cells were then dissociated using Accutase to obtain single cells. PDX media was used to inactivate the Accutase, and dissociated cells were passed through a sterile 70 μm cell strainer (Fisherbrand cat. No. 22363548, Pittsburgh, PA, USA). Cells were then pelleted using a centrifuge at 800–1000 rpm. Accutase-containing media was aspirated, and the cells were resuspended in PDX media and counted using the Countess II FL (Life Technologies Holdings Pte Ltd., Marsiling Industrial Estate #07-06, Singapore).

To minimize edge effects in the plates, we only used the inner 60 wells of the 96-well plate to seed the cells for CFA. The outer 36 wells were filled with 1× PBS or distilled water. To determine plating efficiency, cells were seeded at 50 to 1000 cells per well using 75–100 μL media per well for seeding. After seeding the cells, experimental plates were maintained in a humidified CO_2_ incubator for 24 h. For drug studies, a 2× drug in media solution was prepared and added to the wells containing cells in a 1:1 ratio to achieve the final 1× drug concentration in their respective wells. DMSO was the vehicle control in 0 μM drug wells for our studies. Then, 1–2 h following drug exposure, the plates were taken for irradiation which was performed using one of the following: (1) a conventional X-ray irradiator (Kimtron, Woodbury, CT, USA Medical IC-320 irradiator with 1 mm Cu including 0.1 mm Cu inherent filter at 0.019 Gy/s using 320 kV and 10 mA); (2) Varian ProBeam ultra-high dose rate (FLASH) proton irradiation (320 Gy/s); or (3) Varian ProBeam protons using pulsed doses (1 min delay per pulse) (0.03 Gy/s) for “conventional” dose rate proton irradiation (CONV-RT). Following irradiation, sham and treated plates were returned to the CO_2_ incubator for colony/neurosphere formation. In general, plates were left undisturbed to minimize agitation which can promote colony aggregation. For slower-proliferating neurospheres, 50 μL of media was added after 12 days and they were incubated for an additional 7–8 days to allow for neurosphere/spheroid growth. Once they had reached the desired colony size/formation, colony counting was performed with or without fixation as described below.

### 2.4. Staining the Colonies/Neurospheres

Colony staining was performed using Calcein AM at working concentration of 0.15–0.2 μg/mL under aseptic conditions. In brief, a tube containing 50 μg of Calcein AM is first dissolved in 100 μL of DMSO, which works as a stock dye and can be stored at −20 °C for further use. For the working solution of dye, stock dye is further diluted in required fresh stem cell media as a 1:200 dilution according to the volume requirement to produce a final concentration of 2.5 μg/mL. Dye diluted in media is added to wells containing neurospheres/colonies at approximately 1:15 dilution (e.g., 10–15 μL of dye in a well containing 150–200 μL of neurosphere/colony culture media; but for wells containing 200–250 μL media, then use 15–18 μL of diluted dye). For proper staining, the plates are placed in a CO_2_ incubator for 30 min. After 30 min of incubation, imaging of the inner 60 experimental wells of the 96-well plate at 4× magnification on the Cytation 5 was performed using the 465 nm Filter/LED cube (PN 1225001), which has an excitation of 469/35 nm and an emission of 525/39 nm. Colonies were identified using the size cutoff feature in Cytation 5′s Gen5 analysis tool. For experiments shown, 90–120 μm size cutoffs were used for colony/neurosphere counting.

### 2.5. Fixing the Colony in 96-Well Plates

As an alternative to same-day colony counting, fixing of the wells can be performed on the final day of the CFA by adding 4% paraformaldehyde (PFA) in every experimental well of the 96-well plate and keeping the plates at 4 °C for fixation. Following optimization, we routinely used 40 μL of 4% PFA for fixing the wells, which we found to be stable for months at 4 °C. This approach was most used for tumor lines that were already tagged with a fluorochrome (e.g., GFP or RFP). Without prior labeling, colonies could also be labelled post-fixation using WGA conjugates (see below). Colony counting was performed as in Section 2.4 using size cutoff thresholds in the appropriate fluorochrome channel.

### 2.6. Labelling Colonies with WGA Conjugates

Working WGA conjugate solution was prepared by diluting 1 mg/mL stock of WGA conjugate to a concentration of 20 μg/mL in PBS. For each experiment well containing colonies/neurospheres, which was prefixed with PFA as described earlier in Section 2.5, a volume of 50 μL of the diluted WGA conjugate (20 μg/mL) was added and incubated for 48 h at room temperature for proper staining of the plasma membranes of the colonies’ cells. Stained plates could be kept at 4 °C until imaging/counting as in Section 2.4. Colonies stained with WGA conjugates Alexa Fluor 488 were also imaged with same LED cube (465 nm LED cube) as used during Calcein AM.

### 2.7. Statistical Analysis

GraphPad Prism (v. 10) was used to perform all statistical analysis. Ordinary one-way ANOVA and d-way ANOVA were used in different experimental analyses as discussed in the figure legends. A minimum of 3 replicates were used for all the statistical analysis. Clonogenic survival (semi-logarithmic) curves were generated using the surviving fraction (SF) equation: (number of colonies formed/number of cells plated)/(number of colonies from the sham-irradiated group/number of cells plated) with standard error of the mean (SEM) error bars.

## 3. Results

### 3.1. Clonogenic Assay Using Calcein AM Labeling for Automated Colony Counting

**Figure 1:**
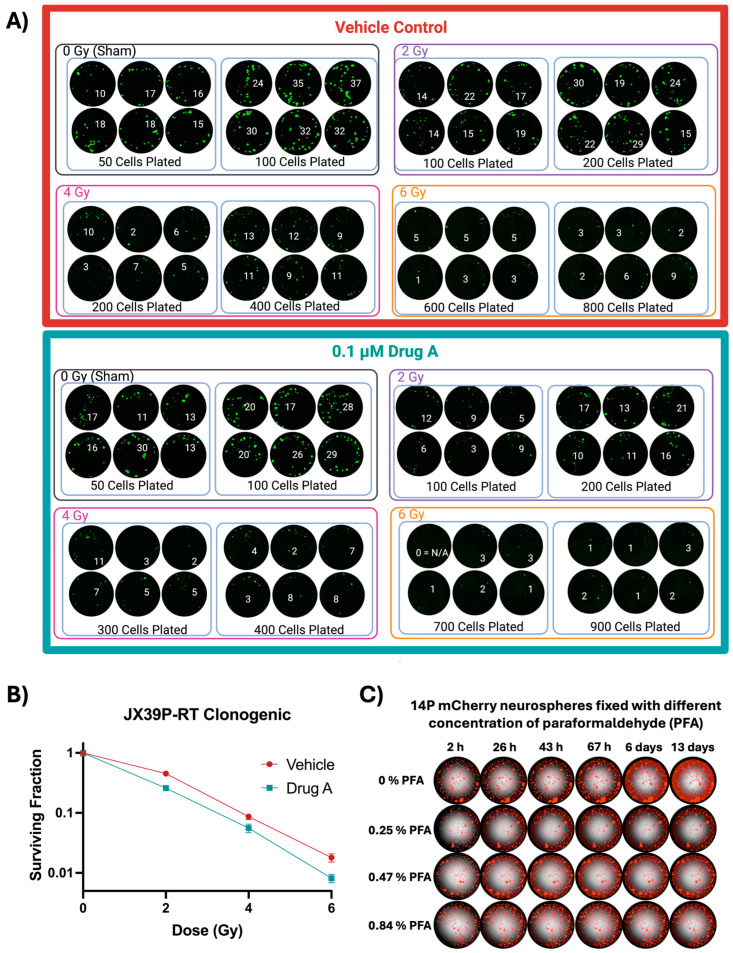
(**A**) 39P-RT cells were seeded in the inner 60 wells of a 96-well plate at different indicated cell numbers and treated with DMSO (vehicle) or Drug A and after 2 h were irradiated with different doses of radiation. After 12 days of colony formation, colonies were stained with 0.2 μg/mL of Calcein AM (green) and imaged at 4× magnification on the Cytation 5, using 120 μm as the cutoff value for colony counting. Colony numbers are indicated in white in each well. (**B**) The semi-log clonogenic curve was plotted with standard error bars. Only the 2 Gy dose was significantly different between Drug A and vehicle (*p* < 0.01) after analyzing two-way ANOVA. (**C**) 14P mCherry neurospheres suspension was used to seed a clear bottom 96-well black-wall plate, and after two days of incubation in a CO_2_ incubator at 37 °C, these neurospheres were fixed with 0, 0.25, 0.47, or 0.84% PFA and then stored at 4 °C. Serial imaging was carried out using the Cytation 5 Imager from 2 h to 13 days using the 590 nm Filter/LED cube (PM 1225002), which has an excitation of 586/15 nm and an emission of 647/57 nm. Created in BioRender.com.

To establish a clonogenic/CFA assay for 3D spheroid cultures, we developed a 96-well plate-based format and performed clonogenic assays using our standard serum-free (BTIC or PDX) neurosphere culture media. Following initial plating efficiency determination, a clonogenic assay was performed using Calcein AM labeling to distinguish viable colonies from cell debris. Colonies were quantified using Gen5 software version 3.15 following imaging of the entire plate on the Cytation5 after a 12-day incubation post-radiation. DMSO vehicle control was compared to 0.1 μM treatment with “Drug A”. Calcein AM-positive objects > 120 μm in diameter were counted as colonies and are indicated in each of the wells shown in Figure 1A, with the clonogenic assay curve shown in Figure 1B.

### 3.2. Standardizing Fixation Protocol with Paraformaldehyde (PFA) for Delayed Counting

Traditional clonogenic/CFA assays include fixation and staining steps that facilitate counting of colonies, which can be performed almost any time afterward. Because neurospheres are non-attached, any delayed imaging would require a fixation process. To determine an appropriate fixation approach, we used mCherry-labeled GBM neurosphere suspensions for seeding in a 96-well plate. After 2 days, neurospheres were fixed with various concentrations of PFA and were stored at 4 °C. Images were taken at various time points to determine the mCherry retention, further neurosphere growth, and in-well positional displacement (Figure 1C). We found that the neurospheres fixed at 0.84% PFA had minimal positional movement and virtually no mCherry bleed out with respect to time. However, we did note neurosphere growth at 4 °C in the wells without fixative (0% PFA) over the 13-day serial imaging. Based on these results, we selected 0.8–0.9% PFA for subsequent fixing of the neurosphere colonies.

### 3.3. Drug Combinations with Radiation

BTIC cultures derived from PDX maintain a more heterogenous cancer cell population as compared to standard immortalized cancer cell line cultures [24,25]. However, with the increasing in vitro passaging of PDX lines, heterogeneity and cancer stem-like cell proportion is diminished, which can be avoided by re-passaging them in immunocompromised athymic nude mice [26]. These advantages of BTIC cell culture over immortalized cell culture make it a promising model in drug and radiation toxicity preclinical testing [27,28]. The drugs, which can also kill BTICs, have high therapeutic value. We next sought to test drug combinations with or without irradiation. Similar in vivo studies are very difficult to perform, especially in initial dose-finding studies.

**Figure 2:**
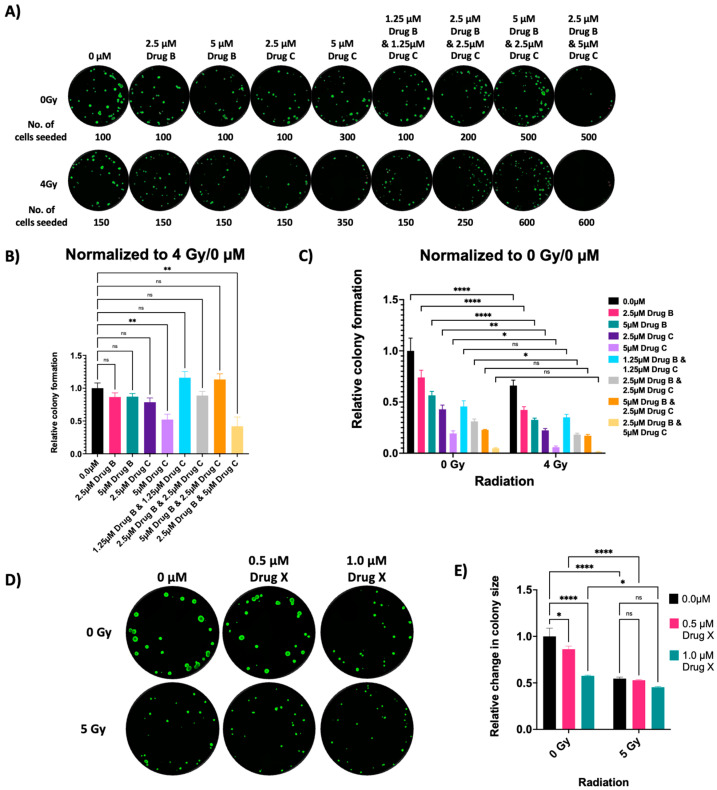
(**A**) 14P NLS mCherry cells were seeded in the inner 60 wells of two 96-well plates. After 24 h, cells were treated with drug B, drug C, and their combination. After 2 h of drug treatment, one plate was irradiated with 4 Gy while the other was sham irradiated. After 12 days of colony formation, colonies were stained with 0.2 μg/mL of Calcein AM (green) and imaged at 4× magnification on the Cytation 5. Colony counts were made using a 100 μm size cutoff. (**B**) Surviving fraction was calculated for each drug/drug combination condition followed by normalization with the 4 Gy/0 μM condition (ordinary one-way ANOVA statistics are shown with SEM). (**C**) Relative colony formations are plotted with normalization only to the 0 Gy/0 μM condition (two-way ANOVA is used for statistical analysis). For (**B**,**C**), * indicates *p* value < 0.05; ** indicates *p* < 0.01; **** indicates *p* < 0.0001; ‘ns’ stands for non-significant. (**D**) 14P BTIC cells were plated at 50 cells/well in 96-well plates and allowed to grow as neurospheres for 5 days. After 5 days, neurospheres were treated with drug X, and after 5 h, they were irradiated with sham (0 Gy) or 5 Gy of radiation. After 12 days of colony/neurosphere formation, colonies were stained with 0.2 μg/mL of Calcein AM (green) and imaged at 4× magnification on Cytation 5. (**E**) Relative change in colony sizes is plotted and two-way ANOVA is used for statistical analysis. * indicates *p* value < 0.05; **** indicates *p* < 0.0001; ‘ns’ stands for non-significant.

The BTIC line 14P was infected with lentivirus expressing a nuclear mCherry (NLS mCherry) label. These 14P NLS mCherry cells were used to seed wells in a clear-bottom 96-well black-wall plate at different numbers according to drug concentration and radiation sensitivity, as shown in Figure 2A–C). After 24 h of seeding, experimental wells of the plates were treated with indicated concentrations of drug B, drug C, or their combination. After 2 h of drug incubation, the plates were irradiated with sham (0 Gy) or 4 Gy. After 12 days of growth, colonies were stained with Calcein AM and imaged at 4×, as in Figure 1. There are two different normalizations shown in order to illustrate drug, drug combination, and radiation interactions. Figure 2B shows the relative colony formation for each drug condition with surviving fractions normalized to 4 Gy/0 μM while Figure 2C shows relative colony formation when simply normalized to 0 Gy/0 μM.

### 3.4. Drug and Radiation Effect on Small Neurospheres (5 Days Grown)

In addition to colony formation/clonogenic assays, our assay also works well in determining the impact of drug and radiation on established spheroids from PDX. Tumor spheroids closely resemble in vivo tumors [24,29], their 3D and compact structure can limit the oxygenation and drug diffusion process, similar to intact tumors [30], and they are suitable for radiation studies [31]. Here, we have used unlabeled 14P GBM BTICs to study the effect of one of our drugs of interest on established spheroids. We seeded 50 cells/well in 96-well plates and allowed small neurospheres to form over 5 days. After 5 days, neurospheres were treated with 0 μM, 0.5 μM, and 1.0 μM of Drug X. After 5 h of drug treatment, neurospheres were irradiated with sham (0 Gy) or 5 Gy. After 7 days post-radiation, colonies/neurospheres were evaluated based on size rather than colony number, as shown in Figure 2D,E). Drug X was potent on its own but did not show radiosensitization.

### 3.5. Conventional and FLASH Radiation Comparison

Clinical radiation and almost all preclinical radiation are delivered at dose rates of <2 Gy/minute, so-called conventional dose rate irradiation (CONV-RT). Ultra-high dose rate (>40 Gy/s), also called FLASH irradiation (FLASH-RT), is an innovative strategy with the potential to improve the therapeutic index for GBM and other cancers. FLASH-RT has been investigated in earnest over the last several years, with several publications coming from the Vozenin lab in Lausanne, Switzerland. FLASH-RT’s effect has predominantly been demonstrated in vivo, and prevailing theories suggest that FLASH-RT rapidly depletes (160 mm Hg/s) oxygen from the cells and tissue, thereby leading to lower reactive oxygen species (ROS) generation and consequently less toxicity to normal cells in comparison to CONV-RT, which depletes oxygen at a much lower rate (0.02 mm Hg/s) [32]. Moreover, the oxygen replenishment rate through oxygen diffusion that corresponds to oxygen consumption rate (2–7 mm Hg/s) is negligible as compared to oxygen depletion (160 mm Hg/s) during FLASH-RT, thus making the irradiated tissue anoxic, which inhibits ROS generation and its detrimental effects to normal tissue, such as resident stem cells [32]. Montay-Gruel et al. also showed that the sparing effect of normal tissue in FLASH-RT is diminished in the presence of carbagen, a chemical which makes tissue more oxygenic [33]. However, some reports established that partial oxygen deficient conditions (e.g., 5% oxygen) promotes normal tissue sparing with efficient antitumor effect by FLASH-RT [34,35].

**Figure 3:**
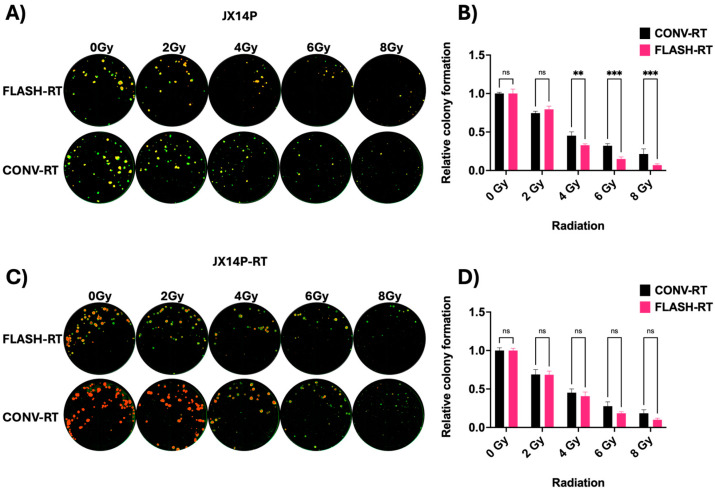
(**A**,**C**) J14P and J14P-RT mCherry, 300 cells/well, were seeded in 96-well plate, and after 24 h, were irradiated with different doses of radiations (0, 2, 4, 6, and 8 Gy). After 13 days of colony/neurosphere formation, colonies were fixed with 0.84% PFA, stained with 4 μg/mL of WGA Alexa Fluor 488 (green), and imaged at 4× magnification using both green and red channels on the Cytation 5. Colonies of size 120 μm or above were counted as true colonies. (**B**,**D**) Relative colony formations are plotted and two-way ANOVA is used for statistical analysis. ** indicates *p* < 0.01; *** indicates *p* < 0.001; ‘ns’ stands for non-significant.

Here, we tested FLASH- and CONV-RT treatment on whole-cell mCherry-labeled 14P and our previously published in vivo radiation selected 14P-RT GBM BTIC lines and performed a CFA in 96-well plates, and the results are shown in Figure 3. The results of CFA show that JX14P were particularly sensitive to FLASH-RT at higher doses (4, 6, and 8 Gy) compared to CONV-RT. However, we observed equi-sensitivity in the JX14P-RT GBM line.

### 3.6. Longitudinal Assessment of Fractionated Radiation Experiment

**Figure 4:**
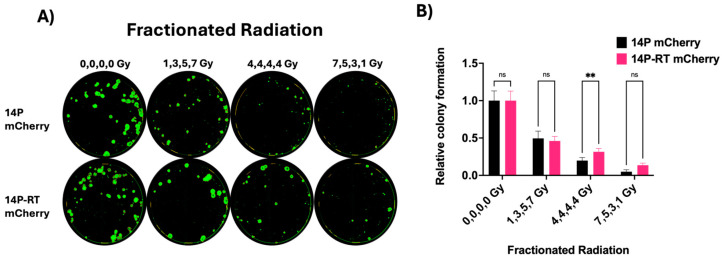
(**A**) 14P mCherry and 14P-RT mCherry neurospheres were plated at 300 cells/well in 96-well plates and irradiated with different radiation doses in a fractionated approach (4 days between fractions). Following fixation with 0.84% PFA, cells were stained with 4 μg/mL of WGA Alexa Fluor 488 (green). (**B**) Relative colony formations are plotted and two-way ANOVA is used for statistical analysis. ** indicates *p* value < 0.01 and ‘ns’ stands for non-significant.

Both single dose and fractionated dose therapy are being utilized in radiation treatment of cancer. However, it is difficult to model multi-fraction therapy in vitro. Here, we have successfully used our CFA to study fractionated radiation treatments on the GBM BTIC line 14P and its acquired radiation resistant line (14P-RT), which were both mCherry-labeled using lentivirus. 14P mCherry or 14P-RT mCherry GBM lines were seeded in four 96-well plates at 300 cells/well. Multi-fraction radiation course of 1, 3, 5, and 7 Gy, 4, 4, 4, and 4 Gy, 7, 5, 3, and 1 Gy, and sham (0, 0, 0, and 0 Gy) were used to irradiate the plates with a 4-day gap between fractions. After the final dose of radiation, colonies were allowed to grow for an additional 5 days and then fixed with 0.8% PFA. Colonies were stained with 4 μg/mL of WGA Alexa Fluor 488 (green). Control colonies of 0, 0, 0, and 0 Gy were fixed on the final day of radiation to avoid overgrowth of colonies. Fixed and stained colonies were imaged at 4× magnification on the Cytation 5 imager. Representative images and their analysis are shown in Figure 4.

## 4. Discussion

GBM is one of the most highly aggressive cancers, and patients with GBM have only 12–15 months of average survival following diagnosis. At present, standard of care for GBM patients include safe surgical resection, radiation therapy, and temozolomide treatment [4]. Although the FDA has approved other drugs in addition to temozolomide, none are as effective as temozolomide. Unfortunately, much of the preclinical drug discovery efforts for GBM have suffered from poor translation to the clinic, potentially because the immortalized GBM cell lines are highly selected for growth and susceptible to drugs that may have either very little or no effect in patient tumors [24,36]. Similarly, combination studies using drugs and radiation have suffered a similar fate. To overcome these limitations and to better represent the heterogenous nature of GBM, patient-derived models of GBM have emerged, including murine-implanted patient-derived xenografts (PDX) and various culture models, including self-aggregating spheroids [26,29,37]. Our group has favored these PDX models as they closely resemble patients, and we have shown that they can be selected for acquired radiation resistance that appears to be representative of clinical radiation resistance, at least at the DNA level [22]. As such, PDX can serve as a valuable tool to better understand GBM biology. In our study, we have exclusively used PDX-derivative models, taking advantage of the fact that PDX lines are highly enriched in cancer stem cells and can be successfully grown in stem cell media (PDX media). However, how best to adapt this culture technique to radiobiological assays is unclear.

The clonogenic assay has remained a critical in vitro assay for radiation-related studies. While high-throughput approaches have been developed for these colony formation assays for immortalized cell lines, adapting these strategies to stem-cell compliant cultures, such as spheroids, has been difficult to implement. GBM patient-derived cells are enriched with cancer stem-like populations (BTICs), which have been shown to survive radiation treatment in vivo and are thought to represent the tumor cells that drive recurrent disease, which is inevitable [38]. Therefore, we pursued our line of experimental design to develop a high-throughput colony formation assay (HT-CFA) to directly address BTIC radiation responsiveness. Our results indicate that 96-well plate formats are suitable for clonogenic assays and can be performed in the absence of scaffolds, matrix material, or other embedding strategies. Furthermore, we have established a simple imaging protocol using the readily available Calcein AM reagent as well as a flexible fixation strategy to facilitate delayed quantification. We have also shown that multi-drug comparison studies with and without radiation are feasible.

Tumor stem cell culture approaches have been garnering interest for many years now and have moved to the forefront of preclinical models for cancer. Indeed, the hypothesis that a stem cell population can tolerate drug and/or radiation treatment to promote the relapse of cancers is not new [39,40]. Yet one of the key features of stem cell culture is the capacity for anchorage-independent growth [41], which complicates clonogenic survival assessment which has historically relied on 2D culture strategies. Nevertheless, CFA in 3D suspension cultures is a staple in vitro stem cell assay, particularly when the extreme limiting dilution assay (ELDA) version is utilized [42]. However, ELDA has a major drawback when testing multiple conditions in that it requires an entire multi-well plate for each sample. Matrix-embedded 3D cultures are also utilized for stem cell CFA [43] but may have cost limitations depending on the matrix that is used and the number of conditions. Soft agar colony assay is a widely used method for spheroid culture [20,21], but it too has certain limitations, including the tedious and time-consuming set-up, which is temperature-sensitive. Several scaffold-based solutions have been developed and some even adapted for radiation-related CFAs, including the 3D microwell CoSeedis matrices [44] and the Alvetex scaffolds [19]. While these approaches are promising and seem to show considerable utility, there can be significant costs for these scaffolds, and most groups still perform these studies using serum-containing medias that are expected to differentiate the cells and have less relevancy.

Our 96-well HT-CFA offers advantages over the other methods discussed above. It is a highly convenient, time-saving, and a cost-effective way to make colonies, even for larger scale studies. For example, we can use a maximum of 60 wells (excluding the outer 36 wells to eliminate edge effects) of a 96-well plate as experimental wells, which is quite sufficient for screening multiple conditions with adequate technical replicates at the same time. In addition, because of the small well size, it is fairly easy to image each well at 4× magnification in four stitched montages to obtain a single composite well image for each well with the Cytation 5. Image acquisition time is reasonable, at 30 to 40 min to capture all 60 wells of a 96-well plate and only a few additional minutes to perform colony calculations. An additional benefit of using a 96-well plate is the minimal media movement during plate handling, greatly limiting cell aggregation, which can easily confound colony counting. Limitations of our approach include the need for an imaging platform, such as the Cytation 5, and the fact that in our hands, a few PDX-derived lines do not form tightly packed spheroids in our culture media. Using a dye such as Calcein AM can distinguish viable colonies from cellular debris but still requires separation of colonies to provide an accurate count. Since the approach relies on counting the colonies and not the level of signal intensity, fluorophore heterogeneity is less of a concern. Stable cell-labeling strategies, as we have shown, are particularly useful for more longitudinal assessment, though variations in labeling intensity can occur across colonies.

Reliable and relevant approaches are desperately needed for radiobiological testing of modern cancer models. Overall, our study provides some practical ways of assessing radiation-related clonogenicity in serum-free spheroids using GBM BTICs derived from PDX. This method could likely be extended to other cancer types capable of making spheroids.

## 5. Conclusions

We have developed a robust and high-throughput 96-well CFA compatible with serum-free cultures of patient-derived self-aggregating tumor neurospheres (BTICs). This approach allows for testing various drug combinations with ionizing radiation in more clinically relevant patient-derived models, such as BTICs. Future directions include the use of robotic multi-dispenser systems to seed the cells in a microwell plates with coupled liquid handlers for more automation. In addition, this assay can be quickly adapted for use in other cancer models that can form spheroids.

## Data Availability

Original raw data is available upon request.
